# Postoperative screening for distress symptom cluster in patients with pituitary adenomas: a propensity-score matched outcome analysis

**DOI:** 10.3389/fneur.2026.1841767

**Published:** 2026-05-08

**Authors:** Dan Wang, Qian Zhang, Ru Ling, Zhizhi Li, Xiling Xia, Haiyan Qiu

**Affiliations:** Department of Neurosurgery, The Affiliated Brain Hospital of Nanjing Medical University, Nanjing, China

**Keywords:** fatigue, pain, pituitary adenomas, quality of life, return-to-work, sleep disturbance

## Abstract

**Objective:**

To evaluate the efficacy of postoperative screening for distress symptom cluster in patients undergoing surgery for pituitary adenoma (PA).

**Methods:**

Prospectively collected data of 334 patients were reviewed. Patients undergoing screening were included in screening cohort. A 1:1 propensity-score matching was applied to select controls. Primary endpoint was Patient Reported Outcomes Measurement Information System (PROMIS) scores for pain interference, fatigue and sleep disturbance at 1 month after surgery between two cohorts. Minimally important difference (MID) was set at −2. Secondary outcomes included inflammatory cytokines, cumulative incidence of hospital readmission or failure return-to-work (RTW) and European Quality of Life Five-dimension (EQ-5D) scores.

**Results:**

Patients in screening cohort were characterized by moderate pain with mild fatigue and sleep disturbance (14.4%), moderate sleep disturbance with mild fatigue (10.8%) and moderate fatigue (8.9%). At 1-month follow-up, mean difference in PROMIS T-scores between the screening cohort and control cohort was −4.926 (*p* < 0.001) for pain interference, −5.283 for fatigue (*p* < 0.001) and −4.475 for sleep disturbance (*p* = 0.038), all exceeding the MID. Compared to controls, patients undergoing screening exhibited lower levels of inflammatory cytokines (all *p* < 0.05), lower cumulative incidences of hospital readmission (log-rank *p* < 0.001) or failure RTW (log-rank *p* < 0.001) and greater levels of EQ-5D scores (all *p* < 0.05) within 6 months after surgery. Minor adverse events occurred in 7.8% of screening cases.

**Conclusion:**

Screening for distress symptom cluster effectively decreased pain interference, fatigue and sleep disturbance after surgery. It also correlated with improved inflammatory status and quality of life, along with a better prognosis regarding hospital readmission or failure RTW during a 6-month follow-up.

## Introduction

Pituitary adenomas (PAs) are usually benign tumors of anterior pituitary that present with characteristic symptoms caused by excess hormone secretion or mass effect from tumor growth (1). It is the third common type of primary brain tumors, accounting for 10 to 15% of all cases. The incidence of PAs was approximately between 3.9 and 7.4 cases per 100,000 per year in general population according to epidemiology data (2). PA can be classified based on their clinical secretory activity. Functioning adenomas (FAs), which secret growth hormone (GH), prolactin (PRL), thyroid stimulating hormone (TSH) or adrenocorticotropic hormone (ACTH), cause acromegaly, amenorrhea-galactorrhea, secondary hyperthyroidism, and Cushing’s disease. In contrast, nonfunctioning adenomas (NFAs) can compress pituitary and surrounding structures, leading to visual impairment or hormone deficiencies (3). Besides the distinctive clinical symptoms caused by hormone secretion or tumor compression, distressing symptoms of fatigue, weakness, pain, or sleep disturbance were often reported in patients with PAs (4). As the first-line treatment for most PAs, transsphenoidal or transcranial surgery can effectively eliminate tumor and reverse symptoms. With the recovery of pituitary function, symptoms associated PAs were significantly alleviated after surgery (5). According to recent studies, distress cluster symptoms such as pain, fatigue and sleep disturbance are one of the most commonly reported symptoms by these patients during the perioperative period. These cluster symptoms can continue for up to 12 months after surgery, leading to a significantly reduced activity and diminished quality of life (6). Although there is currently no established guideline, various non-pharmacological or pharmacological interventions have proven effective in managing this symptom cluster based on evidence from meta-analysis (7).

Given its clinical importance, screening for distressing symptoms was developed to enable refined treatment approaches in our hospital. This study aimed to evaluate the efficacy of postoperative screening for distress symptom cluster on clinical outcomes among patients with PAs.

## Materials and methods

The propensity score-matched (PSM) analysis based on a prospective observational study was conducted in accordance with the Declaration of Helsinki and the Strengthening the Reporting of Observational Studies in Epidemiology (STROBE) guidelines (8). Ethical approval was provided by the institutional Ethics Committee (NBH-2026002). Written informed consent was obtained from all patients.

The study included patients who underwent primary surgery for PAs from January 1, 2023 to December 31, 2025. Patients were eligible if the following criteria were met: (1) a confirmed diagnosis of PA according to the Endocrine Society Clinical Practice guideline (9); (2) indications for transsphenoidal or transcranial surgery (10, 11); (3) American Society of Anesthesiologists Physical Statues I-III; (4) ≥18 years of age. Exclusion criteria were recurrent PA, previous pituitary surgery, other malignant tumors, primary hypothyroidism, primary adrenal diseases, severe nasal disorders, severe hepatic or renal dysfunction, primary psychiatric disorders psychological disorder, alcohol or drug abuse, pregnancy/lactation and incomplete data.

Within 1 week after surgery, patients received either screening for distressing cluster symptom or non-screening based on the decision that most closely aligned with their values and preferences. The screening cohort underwent a pain interference-fatigue-sleep disturbance screening during a face-to-face clinical interview lasting 5 min for each session. Symptoms were measured by the Patient Reported Outcomes Measurement Information System (PROMIS) Profile-29 v2.1. The cutoff point for each of symptom scores was a mean of 50 with a standard deviation (SD) of 10 in reference population (12). Individuals who met the positive criteria were scheduled for a specialized evaluation by a multidisciplinary team consisting of a neurologist, psychologist and rehabilitation specialist. This approach allowed for timely implementation of supports or interventions, such as muscle relaxation, imagery/hypnosis, mindfulness-based stress reduction, cognitive behavioral therapy and pharmacological therapies (non-steroid anti-inflammatory drugs, antiepileptic drugs, weak opioids, sedatives or hypnotics).

To minimize selection bias, the PSM method was used to select patients who did not underwent screening for control at a 1:1 ratio by the nearest matching with a caliper width of 0.2. Propensity scores were calculated using a multivariable logistic regression based on baseline characteristics and clinical covariables: (1) age; (2) sex; (3) race; (4) body mass index; (5) socioeconomic variables; (6) tumor size; (7) tumor functional status; (8) preoperative hypopituitarism; (9) preoperative optic chiasm compression; (10) Knosp classification; (11) Ki-67 and P53; (12) disease duration; (13) comorbidity number; (14) American Society of Anesthesiologists classification; (15) surgical type, scope and duration; (16) perioperative opioids dosage; (17) adjunctive radiotherapy and total dosage; (18) complications; (19) postoperative pituitary insufficiency and hormone replacements.

### Outcome measurements

Demographic and clinical characteristics were obtained from the EMRs. Follow-up data were collected by specially trained investigators at 1, 3 and 6 months after surgery.

Distressing Symptoms experienced in the past 7 days were measure with three subscales from the PROMIS-29 v2.0. Pain Interference scale was used to assess the interference of pain in daily activities including physical, psychological and social functioning. Fatigue scale was used to evaluate the severity of fatigue, ranging from mild feelings of tiredness to a sustained sense of exhaustion. Sleep Disturbance scale was used to assess subjective sleep quality such as refreshing, difficulty falling asleep, and sleep problems. Each subscale comprised of 4 items, with responses rated on a 5-point Likert scale from 1 to 5. The scale descriptors varied depending on the format of difficulty, frequency, severity and global rating: from “without any difficulty” to “unable to do,” “nerve” to “always,” “not at all” to “very much” and “very poor” to “very good,” respectively. The total score for each subscale was standardized using a T-score metric by the Health Measures Scoring Service, with higher scores indicating more severe symptoms (12). Severity of pain interference was categorized into four levels: normal, mild, moderate and severe corresponding to T-score of <50, 50–59, 60–69 and ≥70, respectively. For fatigue or sleep disturbance, severity levels of normal, mild, moderate and severe were identified by T-score of <50, 50–54, 55–74 and ≥75, respectively (13). Serum levels of C-reactive protein (CRP), interleukin-6 (IL-6), interleukin-1β (IL-1β) and tumor necrosis factor-α (TNF-α) were measured at baseline, 1 month and 3 months following surgery as inflammatory markers. Each blood draw was collected at 8 o’clock A.M (14). The 5-level European Quality of Life Five-dimension (EQ-5D-5L) was employed to evaluate Health-related quality of life (HR-QoL), which consisted of 5 dimensions including mobility, self-care, usual activities, pain/discomfort and anxiety/depression. Patients’ agreement with statements on a 5-point Likert scale, ranging from “no problems” to “extreme problems” (15). Cumulative incidence of hospital readmission was defined as readmission for any event related to PAs or surgery, such as surgery complications, hyponatremia, hypopituitarism, residual tumor or recurrence, within 6 months after surgery. Cumulative incidence of failure return-to-work (RTW) was defined as the proportion of patients who were unable to resume work within 6 months after surgery due to physical health status (16). Additionally, adverse events associated treatments for distressing symptom cluster were also recorded.

The primary endpoint was to compare the PROMIS scores at 1 months between two cohorts. Secondary outcomes included the inflammatory markers, cumulative incidence of hospital readmission or failure RTW and EQ-5D-5L scores within 6 months after surgery.

### Sample size calculation

PASS statistical software, version 19.0 (NCSS, LLC. Kaysville, Utah, USA) was used to calculate sample size. According to the published evidence, the minimally important difference (MID) of mean T-scores was considered relevant difference between groups as following: ≥ − 2 for Pain interference, ≥ − 2 for Fatigue and ≥ − 1 for Sleep Disturbance (17, 18). Drawing on a multidisciplinary expert consensus from a series of cases discussions, the researchers would like to generate a sample size for the study to reject the null hypothesis of equal T-scores, if the true difference between the screening and control cohorts ranges from −4 to −2 with a SD of 10 at 1 month after surgery. To achieve 90% power at a two-sided significance level of 5%, 133 cases per cohort were required. Considering a 20% data loss, the final sample size was increased to 167 per cohort.

### Statistical analysis

Statistical analysis was performed using the R Studio software version 4.2.1, with statistical significance defined as *p* < 0.05. Latent class analysis (LCA) was applied to identify subgroups sharing similar symptom profiles after screening. Kolmogorov–Smirnov *Z* test assessed data normality. Normally distributed data and categorical data were reported as mean ± SD and percentage. Differences between cohorts were compared using Student t test and Chi-squared test. One-way repeated measures analysis of variance (rm-ANOVA) was utilized to compare the repeatedly measured data, followed by *post-hoc* analysis with Bonferroni correction. Cumulative incidence was performed using Kaplan–Meier curves and the log-rank test. Missing follow-up data were addressed using multiple imputation model.

## Results

**Figure 1:**
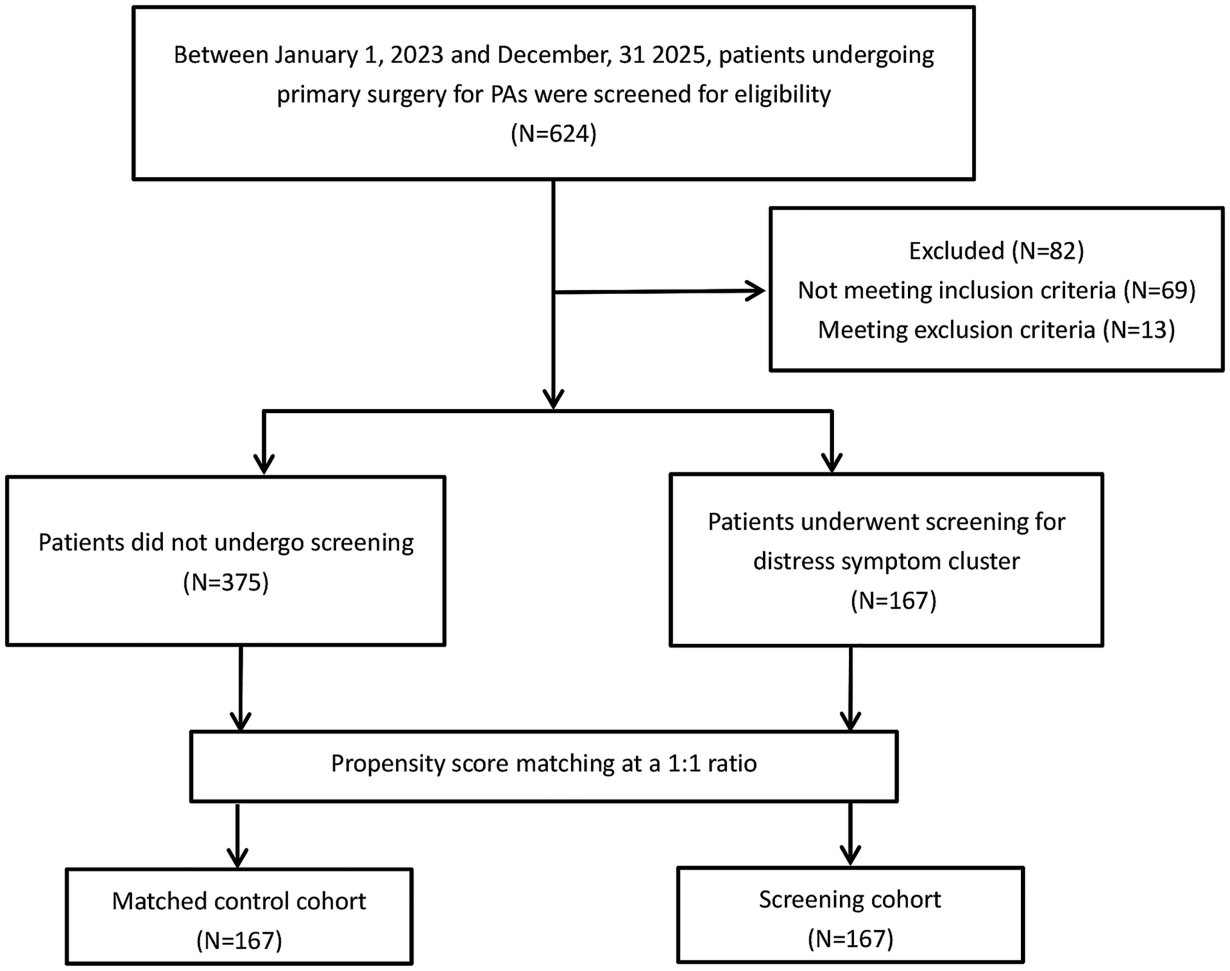
The flow diagram of the study cohorts. PA, pituitary adenomas; PSM, propensity score-matched.

Figure 1 showed the study flowchart. After 1:1 PSM, each cohort comprised 167 patients. Table 1 showed the demographic and clinical characteristics of patients. The standardized mean differences (SMDs) for all covariates were <0.1 following matching, indicating that the two cohorts were well balanced (all *P*>0.05).

**Table 1:** Comparison of demographic and clinical variables in patients undergoing primary surgery for PA.

Variables	Screening cohort (*n* = 167)	Control cohort (*n* = 167)	*t*/*x*^2^ value	*p*	SMD
Age-yr (mean ± SD)	55.67 ± 11.51	56.03 ± 12.29	−0.276	0.782	−0.030
Sex-no. (%)			0.048	0.913	
Female	80 (47.9%)	78 (46.7%)			0.024
Male	87(52.1%)	89 (53.3%)			−0.024
Race-no. (%)			0.414	0.607	
Han Chinese	130 (77.8%)	125 (74.9%)			0.068
Minority	37 (22.2%)	52 (25.1%)			−0.068
BMI-kg/m^2^ (mean ± SD)	23.62 ± 2.53	23.58 ± 3.12	0.129	0.898	0.014
Employment-no. (%)			0.526	0.505	
Independent	60 (35.9%)	63 (32.3%)			0.076
Employment	107 (64.1%)	104 (67.7%)			−0.076
Work status-no. (%)			0.278	0.693	
Physically demanding work	39 (23.4%)	35 (21.0%)			0.058
Mentally demanding work	128 (76.6%)	132 (79.0%)			−0.058
Income status-no. (%)			0.194	0.908	
<5,000¥	34 (20.4%)	36 (21.6%)			−0.029
5,000–10,000¥	89 (53.3%)	85 (50.9%)			0.088
>10,000¥	44 (26.3%)	46 (27.5%)			−0.027
Medical insurance-no. (%)			0.149	0.797	
Out-of-pocket payments	38 (22.8%)	41 (24.6%)			−0.042
Medicare reimbursements	129 (77.2%)	126 (75.4%)			0.042
PA size-no. (%)			0.389	0.823	
Microadenoma	26 (15.6%)	22 (13.2%)			0.068
Macroadenoma	104 (62.3%)	107 (64.1%)			−0.037
Giant adenoma	37 (22.2%)	38 (22.8%)			−0.014
PA functional status-no. (%)			1.428	0.839	
Nonfunctioning	95 (56.9%)	101 (60.5%)			−0.073
Prolactin	25 (15.0%)	24 (14.4%)			0.017
Adrenocorticotropic hormone	21 (12.6%)	23 (13.8%)			−0.035
Growth hormone	17 (10.2%)	13 (7.8%)			0.084
Thyroid stimulating hormone	9 (5.4%)	6 (3.6%)			0.087
Preoperative hypopituitarism-no. (%)	81 (48.5%)	79 (47.3%)	0.048	0.913	0.024
Optic chiasm compression-no. (%)	31 (18.6%)	29 (17.4%)	0.081	0.887	0.031
Knosp classification-no. (%)			0.352	0.986	
Grade 0	28 (16.8%)	31 (18.6%)			−0.047
Grade 1	39 (23.4%)	41 (24.6%)			−0.028
Grade 2	37 (22.2%)	36 (21.6%)			0.015
Grade 3	37 (22.2%)	35 (21.0%)			0.029
Grade 4	26 (15.6%)	24 (14.4%)			0.034
Ki-67-no. (%)			0.372	0.830	
<3%	126 (75.4%)	129 (77.2%)			−0.042
3–5%	29 (17.4%)	25 (15.0%)			0.065
>5%	12 (7.2%)	13 (7.8%)			−0.023
P53-no. (%)			0.660	0.719	
Negative	139 (83.2%)	134 (80.2%)			0.078
Positive	20 (12.0%)	22 (13.2%)			−0.036
Weak	8 (4.8%)	11 (6.6%)			−0.078
Time to diagnosis-months (mean ± SD)	47.92 ± 22.03	48.12 ± 21.99	−0.083	0.934	−0.009
Number of comorbidity (mean ± SD)	2.19 ± 1.16	2.23 ± 1.19	−0.311	0.756	−0.034
ASA classification-no. (%)			0.613	0.736	
I	9 (5.4%)	12 (7.2%)			−0.074
II	77 (46.1%)	79 (47.3%)			−0.024
III	81 (48.5%)	76 (45.5%)			0.060
Operative type-no. (%)			0.114	0.866	
Open surgery	19 (11.4%)	21 (12.6%)			−0.030
Endoscopic surgery	148 (88.6%)	146 (87.4%)			0.030
Operative scope-no. (%)			0.502	0.778	
Partial	12 (7.2%)	14 (8.4%)			0.076
Subtotal	41 (24.6%)	45 (26.9%)			−0.045
Total	114 (68.3%)	108 (64.7%)			0.076
Surgery duration-min (mean ± SD)	157.15 ± 26.78	158.02 ± 25.43	−0.304	0.761	−0.033
Intraoperative remifentanil dosage-ng (mean ± SD)	1232.42 ± 241.97	1236.53 ± 298.37	−0.138	0.890	−0.015
Postoperative sufentanil dosage through PCIA-ug (mean ± SD)	258.12 ± 20.80	257.64 ± 21.22	0.209	0.835	0.023
Adjunctive radiotherapy-no. (%)			0.109	0.869	
No	147 (88.0%)	145 (86.8%)			0.036
Yes	20 (12.0%)	22 (13.2%)			−0.036
Total dosage of radiotherapy-Gy (mean ± SD)	59.04 ± 3.86	58.95 ± 3.79	0.215	0.830	0.024
Postoperative complications-no. (%)					
Epistaxis	27 (16.2%)	24 (14.4%)	0.208	0.761	0.050
Nasal adhesions	22 (13.2%)	25 (15.0%)	0.223	0.753	−0.052
Olfactory disturbance	36 (21.6%)	33 (19.8%)	0.164	0.787	0.044
Transient diabetes insipidus	19 (11.4%)	21 (12.6%)	0.114	0.866	−0.037
Cerebrospinal fluid rhinorrhea	15 (9.0%)	17 (10.2%)	0.138	0.853	−0.041
Hyponatremia	23 (13.8%)	26 (15.5%)	0.195	0.757	−0.048
Postoperative pituitary insufficiency-no. (%)			0.501	0.973	
None	125 (74.9%)	121 (72.5%)			0.055
Cortisol	18 (10.8%)	20 (12.0%)			−0.038
Thyroid hormone	13 (7.8%)	15 (9.0%)			−0.043
Growth hormone	7 (4.2%)	6 (3.6%)			0.031
Sex steroids	4 (2.4%)	5 (3.0%)			−0.037
Postoperative hormone replacements-no. (%)			0.630	0.960	
None	130 (79.3%)	127 (76.0%)			0.079
Glucocorticoids	15 (9.1%)	17 (10.2%)			−0.037
Thyroxine	10 (6.1%)	11 (6.6%)			−0.021
Growth hormone	7 (4.3%)	9 (5.4%)			−0.051
Sex hormones	2 (1.2%)	3 (1.8%)			−0.049

PA, pituitary adenoma; BMI, body mass index; ASA, American Society of Anesthesiologists; PCIA, patient controlled intravenous analgesia; SD, standard deviation; SMD, standard mean difference. Comorbidity included hypertension, diabetes, cardiac disease, cerebrovascular disease, and others.

**Figure 2:**
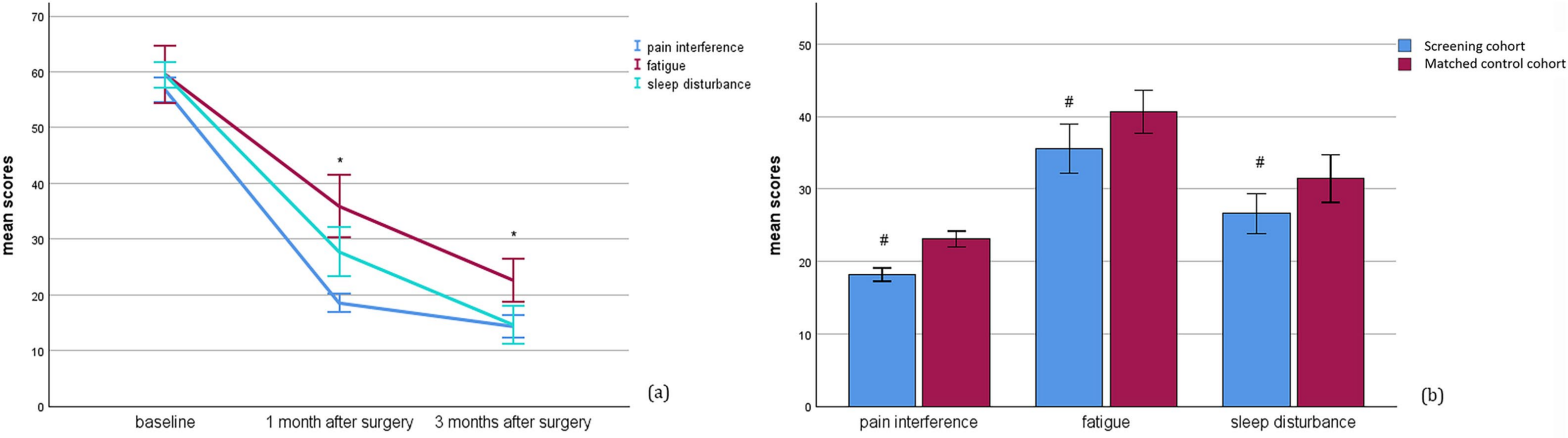
PROMIS outcomes for pain interference, fatigue and sleep disturbance. **(a)** PROMIS T-scores across all time points in the screening cohort; **(b)** mean difference in PROMIS T-scores between the screening cohort and matched control cohort at 1 month after surgery. PROMIS, Patient Reported Outcomes Measurement Information System. ^*^p < 0.017 between time points and ^#^*p* < 0.05 between two cohorts.

According to LCA analysis, patients undergoing screening at 1 week after surgery were divided into four classes based on the types and severity of symptoms. In the screening cohort, 65.9% of patients exhibited all symptoms within normal ranges and were identified as class I. Those with moderate pain accompanied by mild fatigue and sleep disturbance were categorized as Class II (14.4%). Cases characterized by moderate sleep disturbance with mild fatigue fell into Class III (10.8%), while individuals experiencing moderate fatigue only were assigned to Class IV (8.9%) (Table 2). As shown in Figure 2a, mean T-scores for pain interference, fatigue and sleep disturbance in the screening cohort significantly decreased at the 1- and 3-month follow-up visits after receiving interventions post-screening when compared to their baseline values at 1 week after surgery (all *p* < 0.001). At 1 month after surgery, there was a statistically significant difference between the screening and matched control cohorts in mean T-scores for pain interference (18.17 ± 7.68 vs. 23.10 ± 8.15, *p* < 0.001), fatigue (35.57 ± 9.92 vs. 40.85 ± 8.76, *p* < 0.001) and sleep disturbance (26.60 ± 8.056 vs. 31.08 ± 9.69, *p* = 0.038). The mean differences were −4.926 (95%CI: −6.314, −3.538) for pain interference, −5.283 (95%CI: −9.709, −0.856) for fatigue and −4.475 (95%CI: −8.701, −0.249) for sleep disturbance, all of which exceeded the threshold of −2 for MID (Figure 2b).

**Table 2:** Difference in distress symptom cluster in screening cohort according to LAC analysis.

PRMIS item	Class I (*n* = 110)		Class II (*n* = 24)		Class III (*n* = 18)		Class IV (*n* = 15)	
	Mean	SE	Mean	SE	Mean	SE	Mean	SE
Pain interference	14.891	0.430	64.250	0.577	13.722	1.252	14.533	1.333
Fatigue	13.491	0.509	54.083	1.170	52.389	0.295	63.333	0.691
Sleep disturbance	8.764	0.459	52.208	1.311	63.722	0.642	14.600	1.023

Treatment	Number of patients (%)	Number of patients (%)	Number of patients (%)	Number of patients (%)
Physical therapy	0	4 (16.7%)	0	9 (60.0%)
Psychological therapy	0	2 (8.3%)	8 (44.4%)	5 (40.0%)
Pharmacal therapy	0	18 (75.0%)	10 (55.6%)	0

Class 1 = all symptoms within normal ranges; Class 2 = pain with fatigue and sleep disturbance; Class 3 = sleep disturbance with fatigue; Class 4 = fatigue.

LAC, latent profile analysis; PRMIS, patient reported outcomes measurement information system.

**Figure 3:**
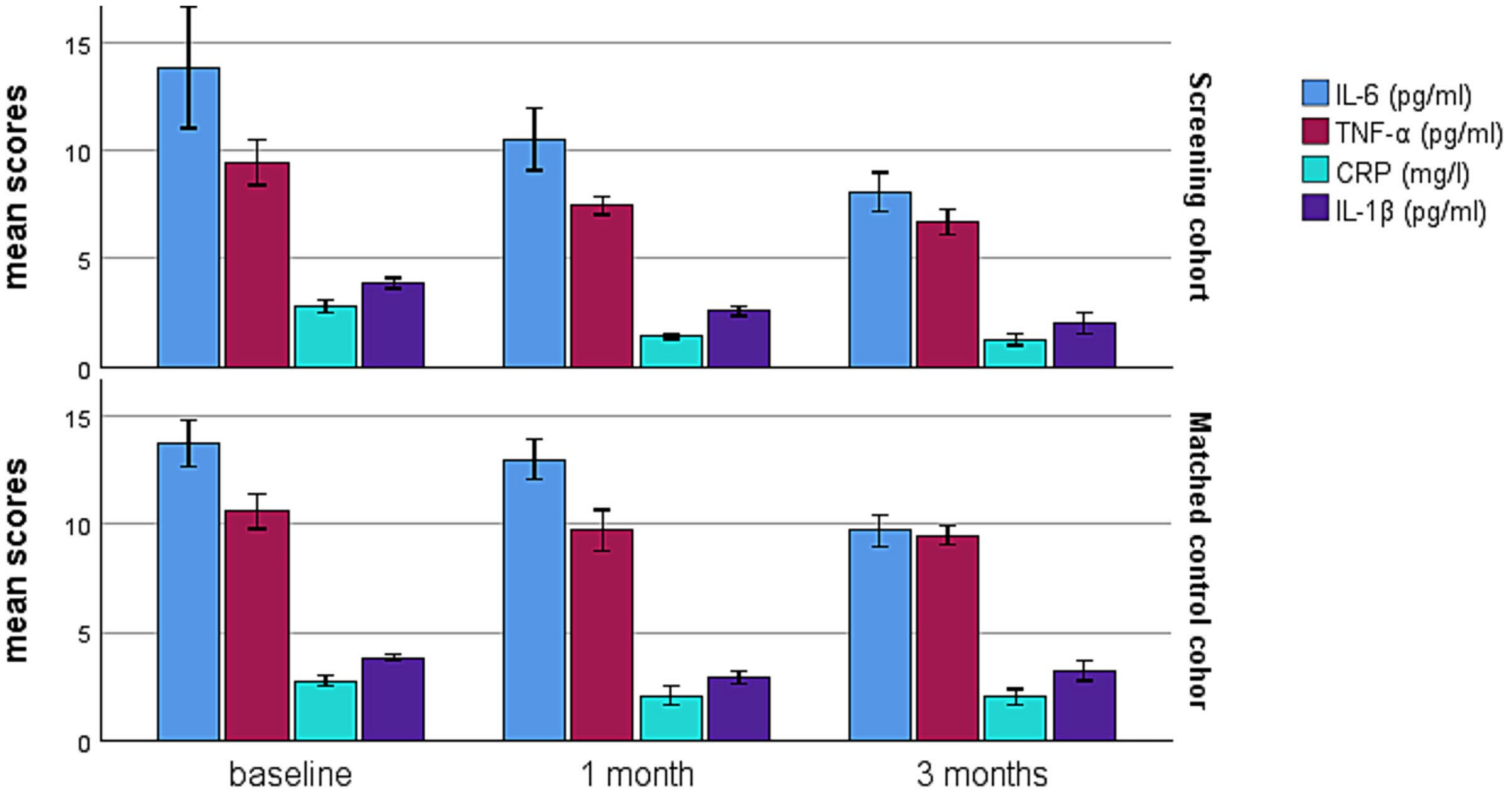
Comparison of inflammatory cytokines between the screening cohort and matched control cohort within 3 months after surgery for PA. PA, pituitary adenomas; IL-6, interleukin-6; TNF-α, tumor necrosis factor-α; CRP, C-reactive protein; IL-1β, interleukin-1β. ^*^p < 0.05 between the two cohorts.

Figure 3 illustrated the reduction in inflammatory cytokines, including CRP, IL-6, IL-1β, and TNF-α from baseline prior to surgery to 3 months post-surgery in both two cohorts. However, lower levels of CRP (1.38 ± 0.65 vs. 2.18 ± 0.57, *p* < 0.001), IL-6 (10.52 ± 2.13 vs. 12.99 ± 1.36, *p* = 0.004) and TNF-α (7.62 ± 1.28 vs. 9.58 ± 1.72, *p* = 0.001) were observed in the screening cohort compared to the matched control cohort at 1 month after surgery. At the 3-month follow-up, the screening cohort continued to have lower levels of CRP (1.29 ± 0.36 vs. 2.09 ± 0.55, *p* < 0.001), IL-6 (7.89 ± 1.33 vs. 9.76 ± 1.08, *p* = 0.002), IL-1β (2.08 ± 0.69 vs. 3.30 ± 0.73, *p* < 0.001) and TNF-α (6.74 ± 1.93 vs. 9.15 ± 1.11, *p* < 0.001) than the matched control cohort.

**Figure 4:**
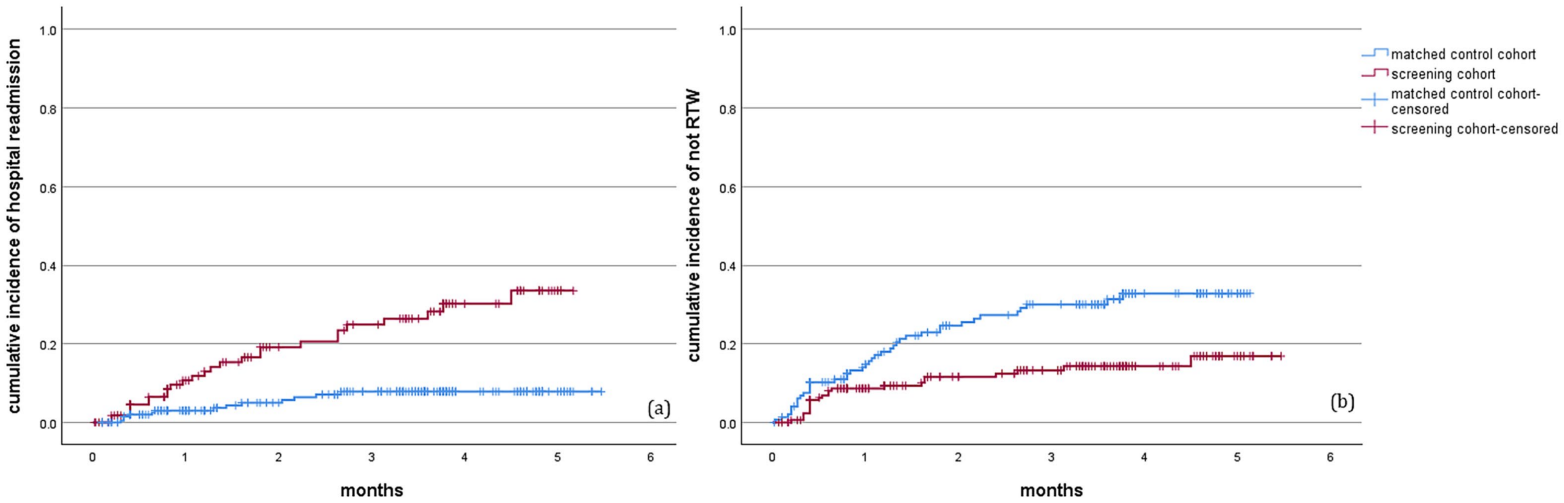
Kaplan–Meier curves for patients between two cohorts within 6 months follow-up. **(a)** The cumulative incidence of hospital readmission was significantly lower in screening cohort than that in control cohort (log-rank test, *p* < 0.001); **(b)** The cumulative incidence of failure RTW was significantly lower in screening cohort than that in control cohort (log-rank test, *p* < 0.001). RTW, return-to-work.

Based on Kaplan–Meier analysis, the cumulative incidence of hospital readmission within 6 months after surgery was 9.1% of patients in the screening cohort, which was lower than that of 21.8% in the matched control cohort (log-rank test *p* < 0.001). Furthermore, the cumulative incidence of failure RTW was lower in the screening cohort compared to the matched control cohort (12.6% vs. 27.5%, log-rank test *p* < 0.001) (Figure 4).

**Figure 5:**
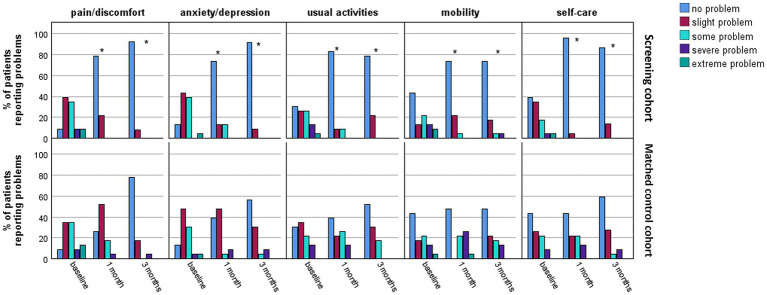
The proportion of patients reporting problems across five domains of EQ-5D-5L during 6 months after surgery for PA. EQ-5D-5L, 5-level European Quality of Life Five-dimension; PA, pituitary adenomas. ^*^p < 0.05 between the two cohorts.

As shown in Figure 5, both two cohorts demonstrated improvements in all five domains of the EQ-5D-5L scores across all time points during follow-up. However, a greater percentage of patients in the screening cohort reported no problems across all five dimensions compared to the matched control cohort at both the 1-month and 3-month follow-ups, with all *p* < 0.001 (Figure 5).

No serious complications related to physical, psychological and pharmacological treatments for pain, fatigue or sleep disturbance were observed in the screening cohort. Minor complications including nausea, vomiting, dizziness and tremor occurred in 7.8% of cases in this cohort.

## Discussion

This study was the first to applied postoperative screening for distress symptom cluster in patients undergoing primary surgery for PA. Our findings showed that the screening combined with specialized treatments significantly decreased the severity of pain interference, fatigue and sleep disturbance after surgery. It was also related to better postoperative outcomes in terms of inflammatory status, hospital readmission, failure RTW and HR-QoL, compared to matched control patients.

Prior review showed that up to 60% of patients exhibited moderate to severe pain following craniotomy. Despite the less invasive nature of transsphenoidal surgery, pain remained a common postoperative complication (19). Furthermore, chronic headache occurred in 33 to 72% of patients with PAs due to several mechanisms. Headache occurrence was significantly associated with tumor size, optic chiasm compression and cavernous sinus invasion. Patients with FAs, particularly GH- or PRL-secreting tumors were more prone to experience headache (20). Wolf et al. (21) found that 70% of patients reported improvement in at least one category of the Headache Impact Test, while 7.6% of cases reported worsening headache after undergoing endoscopic transsphenoidal resection for PAs. According to a review evaluating alterations in diurnal rhythmicity in patients receiving surgery for NFAs, melatonin secretion was often disrupted in these patients (OR = 5.3, 95%CI: 1.9–30.6), leading to a lower sleep efficiency with less rapid eye movement sleep observed in polysomnography and a fragmentation of sleep–wake rhythm with longer sleep duration measured by actigraphy. These complaints might result from the disturbed entrainment or damage of the hypothalamic suprachiasmatic nucleus caused by the suprasellar macroadenoma or its treatment (22). Another hypothesis was that the complaints of sleep dysfunction might be attributed to hypopituitarism, hormonal replacements or apathy syndrome after surgery (23). Regarding to fatigue, a recent longitudinal study involving 196 Chinese patients with PAs showed that patients were divided into no fatigue group (13.2%), fatigue remission group (64.6%) and persistent fatigue group (22.2%) based on the postoperative fatigue trajectories within 3 months after surgery (24). Another prospective study reported that most patients met the criteria of normal fatigue with a mean Multidimensional Fatigue Inventory scores of 50.0 ± 22.4 at 12 months following surgery. However, approximately 30% of patients still reported severe fatigue, which might be associated with hypothalamic function, hormonal status and affective state (4).

According to the cancer symptom management researches, pain, fatigue and sleep disturbance typically do not occur in isolation, two or more of them occurred together to form one of the most symptom clusters in cancer patients, leading to a greater impact on physical function and quality of life (25). In addition, the PROMIS employed the Item Response Theory (IRT) and incorporated Computerized Adaptive Tests (CATs) to develop a standardized and efficient approach for outcome measurements. This approach used a few items to cover multiple dimensions such as pain, fatigue, psychiatric symptoms, sleep disturbance, cognitive function and social functions. It also allowed for comparison across different diseases and patients, overcoming the limitations of conventional questionnaires and even the pituitary-specific quality of life questionnaire (PitQoL), which contained 64 items (26). In alliance with previous data, our results demonstrated that patients after surgery for PAs were divided into four distinct classes at 1 week after surgery for PAs by PROMIS screening: class I, normal symptoms (65.9%), class II, moderate pain with mild fatigue and sleep (14.4%), class III, moderate sleep disturbance with mild fatigue (10.8%) and class IV, moderate fatigue (8.9%). A systematic review and network meta-analysis investigated a variety of nonpharmacological interventions for the pain-fatigue-sleep disturbance symptom cluster, and recommended progressive muscle relaxation and mindfulness-based stress reduction for this distress symptom cluster (27). Pharmacological therapies for pain, fatigue or sleep disturbance were also effective in managing this symptom in clinical practice (28, 29). In this study, patients in the screening cohort reported a significantly lower mean T-score in terms of pain interference, fatigue and sleep disturbance after receiving corresponding treatments, with the mean difference exceeding MID at the 1-month follow-up compared to the matched controls.

Inflammatory cytokines were proved to be related to the symptom cluster of pain, fatigue and sleep disturbance according to results of path analyses. Higher levels of these inflammatory biomarkers indicated greater symptoms, and in turn resulted in greater interference with quality of patients’ daily life (30). Our results showed that higher levels of inflammatory biomarkers, better EQ-5D-5L scores in the screening cohort. Additionally, the 30-day readmission rate and overall rate of RTW were also significantly lower in the screening cohort compared to the matched cohort.

This study had several limitations. Firstly, the outcome assessors were not blinded to cohort assignment. Secondly, patients selected the screening option, resulting in significant self-selection introduces massive inherent bias that could not be completely addressed by PSM. Thirdly, since the control cohort lacked baseline PROMIS scores, it was challenging to exclude initial differences in symptom severity, leading to a risk that the observed improvements might be influenced by regression to the mean, especially if the screening group had higher symptom scores at baseline. Fourthly, RTW was a multifactorial outcome that was often influenced by socioeconomic status, occupational demands, and insurance factors, which might not have been fully accounted for in the matching process. Lastly, the follow-up duration was limited to 6 months, which prevented analysis of long-term outcomes that would strengthen the clinical significance of the study. Future research should include a well-designed randomized controlled trial to validate these results.

## Conclusion

In conclusion, the postoperative screening for distress symptom cluster followed by appropriate treatments significantly decreased patients’ levels of pain interference, fatigue and sleep disturbance. It was correlated with better postoperative inflammatory status and HR-QoL, along with a lower cumulative incidence of hospital readmission and failure RTW over a 6-month follow-up in patients undergoing primary surgery for PAs. Therefore, it was advised to include this screening as a component of routine postoperative care for these patients.

## Data Availability

The data are available from the corresponding author upon reasonable request.

## Glossary

PA: pituitary adenoma
FA: functioning adenoma
NFA: nonfunctioning adenoma
GH: growth hormone
PRL: prolactin
TSH: thyroid stimulating hormone
ACTH: adrenocorticotropic hormone
PSM: propensity score-matched
PROMIS: Patient Reported Outcomes Measurement Information System
EMRs: electronic medical records
IL-6: interleukin-6
TNF-α: tumor necrosis factor-α
CRP: C-reactive protein
IL-1β: interleukin-1β
HR-QoL: health-related quality of life
EQ-5D-5L: 5-level European Quality of Life Five-dimension
RTW: return-to-work
MID: minimally important difference
LCA: latent class analysis
ANOVA: analysis of variance
SMD: standardized mean differences
SD: standard deviation
IQR: inter quartile range
CI: confidence interval
